# Supplementary material for: Estimating sales transitions between competing products via optimal transport
Source: PLoS One. 2025 Jun 6;20(6):e0325173. doi: 10.1371/journal.pone.0325173 (PMC12143541; doi:10.1371/journal.pone.0325173)
Supplement: S1 Appendix — (PDF) [file pone.0325173.s001.pdf]

[Parameters Used in Alcoholic Beverages Experiment]

α : 0.1

β : 0.1

ε : 9e-7

[Parameters Used in Coffee Beverages Experiment]

α : 6e-3

β : 2e-3

ε : 2e-6

[Parameters Used for Fine-tuning]

Learning Rate: 2e-5

Number of Training Epochs: 1

Ratio of Training Data, Test Data, and Validation Data: 4:3:3

Batch Size: 32
