# Supplementary material for: Estimating sales transitions between competing products via optimal transport
Source: PLoS One. 2025 Jun 6;20(6):e0325173. doi: 10.1371/journal.pone.0325173 (PMC12143541; doi:10.1371/journal.pone.0325173)
Supplement: Attachment — Submitted filename: author-response.pdf [file pone.0325173.s002.pdf]

Authors' Responses to the Reviewers' Comments

Title Estimating Sales Transitions between Competing Products via Optimal Transport

Authors Shoki Yamao, Ryota Ueda, Shoichiro Koguchi, Michi Nakase, Aru Suzuki,
Kohdai Toyoda, Ken Kobayashi, and Kazuhide Nakata

We wish to express our appreciation to all the reviewers for their insightful comments, which helped us significantly improve the quality of our manuscript. We have carefully examined the reviewers' comments and revised the manuscript. Please note that, for the convenience of the reviewers, major corrections have been indicated in blue letters in the revised manuscript. Our point-by-point responses to the reviewers' comments are described as follows.

Responses to Reviewer #1

Comment #1 *The abstract could briefly mention the limitations of the study, such as the proprietary nature of the data or potential improvements using customer identifiers.*
Response: Thank you for your insightful suggestion. In response to the reviewer's comment, we have revised the abstract to explicitly mention the study's limitations. Specifically, we acknowledge that the use of proprietary data from the Japanese beverage industry may limit the reproducibility of our results. Additionally, the absence of customer identifiers (IDs) in our dataset prevents us from directly tracking individual customer transitions between products. Incorporating customer IDs in future studies could enable a deeper analysis of customer behavior and improve the robustness of our findings.

Comment #2 *I would suggest including a flowchart or diagram summarizing the methodology for easier comprehension.*
Response: In accordance with the reviewer's suggestion, we have added a figure to give an overview of the proposed methodology in the introduction.

Comment #3 *Heatmaps and transition diagrams are valuable but lack detailed captions. I would suggest that you explain what each axis represents and highlight the key observations in each figure.*
Response: Thank you for your valuable feedback. Following the reviewer's suggestion, we have updated the captions for the heatmaps and transition diagrams in the revised manuscript to clarify how to interpret the figures and highlight the key observations.

Responses to Reviewer #2

Summary Comment #1 *First, I appreciate the opportunity to review this manuscript, which explores the estimation of sales transitions between competing products using an optimal trans-*

port framework. This is a timely and relevant study, particularly in mature markets where understanding customer switching behaviour is critical for companies seeking to maintain market share under increasingly stringent privacy regulations. The manuscript presents an innovative approach by framing sales transitions as an optimal transport problem, complemented by thoughtful regularisations to reflect realistic market dynamics. The integration of a projected gradient method to solve the optimisation problem and its validation with real-world POS data from the Japanese beverage industry adds significant practical value to the study.
Response: We sincerely appreciate the reviewer's thoughtful and positive assessment of our manuscript. We have carefully considered all of the reviewer's comments and have incorporated the suggested revisions wherever possible. We hope that the revised manuscript meets the reviewer's expectations.

Summary Comment #2 *However, while the manuscript offers a promising contribution, certain areas require improvement to enhance its clarity, robustness, and overall impact. The theoretical rationale for framing the problem as an optimal transport task could be articulated more clearly, as this would help establish a stronger foundation for the proposed methodology. Additionally, further justification of the two regularisation assumptions and their implications on market behaviour would strengthen the argument.*
Response: Thank you for your helpful feedback. We have revised the introduction to clarify why we frame the estimation problem as an optimal transport task and explained that this formulation can avoid the trivial solutions that arise in the existing method [2].

Regarding the regularizations, we have added a detailed explanation of the two regularization assumptions and their implications on market behavior in the corresponding part by citing additional references.

Summary Comment #3 *In the methodology section, greater detail regarding the implementation of the projected gradient method, including computational considerations and convergence properties, would enhance the transparency and reproducibility of the results. Furthermore, while the case studies provide valuable insights, deeper discussion on the generalisability of the findings beyond the Japanese beverage industry would improve the study's relevance to broader markets. I hope the authors will find these comments constructive and useful in refining their manuscript for publication.*
Response: Thank you for your important suggestions. For our projected gradient method, we can bound the difference between the objective value of the generated solutions and that of the optimal solution using existing theoretical results [1]. We have added the theoretical analysis of the convergence properties of the projected gradient method to the revised manuscript.

As for the generalizability of our findings, we have additionally included a discussion on the implications for marketing and policy decisions, explaining how our findings can extend beyond the Japanese beverage market. Specifically, we explain that many mature markets share similar challenges—strict privacy regulations, limited overall growth, and intense competition among major brands—and that our approach to modeling changes in consumer behavior (including policy revisions and new product launches) can be adapted to these contexts. We also clarify how analyzing tax-based shifts in purchasing helps policymakers and companies plan for demand fluctuations in various environments.

Comments #1 *The introduction provides a clear context for the study and highlights the importance of understanding customer transitions in mature markets. However, the articulation of research gaps could be more robust. While the authors note the increasing difficulty of obtaining purchase history data with customer identifiers due to privacy regulations, this is not sufficiently*

linked to broader gaps in the existing literature. For instance, the discussion on previous studies (lines 22–42) largely summarises methodologies without critically addressing their limitations in terms of applicability, scalability, or assumptions in real-world contexts. Moreover, while the authors mention challenges with the trivial solution in Chiba et al.'s linear programming method, there is insufficient discussion on why these limitations necessitate the proposed optimal transport framework and how it uniquely addresses unresolved questions in the field. Strengthening the argument for the novelty and necessity of this study would enhance the introduction significantly.

Response: Thank you for your insightful comments. According to the reviewer's suggestion, we have significantly revised the introduction to highlight the research gaps and the necessity of our proposed optimal transport framework. First, we include a discussion that explains that the previous brand-switching studies often rely on data tied to specific customers or survey responses—datasets that are increasingly difficult to collect due to privacy regulations. In doing so, we contrast these works with Chiba et al. [2], which does not require customer IDs but can produce trivial solutions even when market shares shift.

Next, we explain why these trivial solutions are problematic, given that real markets naturally involve a certain level of switching whenever product shares change. By examining the limitations of these existing methods, we motivate the use of an optimal transport framework, which naturally avoids the trivial solution. We believe this clarification strengthens the argument for our study's novelty and usefulness, especially in settings where privacy constraints render customer-level data unavailable.

Comment #2 *The manuscript lacks a dedicated literature review or theoretical foundation section between the introduction and methodology. While the introduction briefly summarises prior work on brand-switching and related methods (lines 22–42), this does not sufficiently review the broader body of literature or establish the theoretical underpinnings of the study. A standalone section could elaborate on key concepts such as optimal transport theory, brand-switching behaviour, and market dynamics, providing deeper insight into how these areas interconnect. Additionally, a comprehensive discussion of relevant studies, including their contributions and limitations, would better contextualise the research gap and justify the novelty of the proposed method. This would strengthen the foundation for the study and enhance its academic contribution.*

Response: Thank you for the constructive suggestion. We have expanded our discussion of related literature and theoretical foundations by creating a more substantial part in the introduction, ensuring that our study is clearly situated within the broader research context. In this expanded section, we categorize existing studies into those that rely on customer IDs or surveys and those that use only aggregated purchase data. While the former can yield detailed insights, we explain that these approaches often pose challenges in terms of data availability and heightened privacy regulations.

For studies that do not require customer identifiers, we provide an in-depth review of Chiba et al. [2], elaborating on why their method can produce meaningless estimates even when market share shifts occur and how that limitation motivates the use of our optimal transport framework. We also provide an overview of optimal transport theory, highlighting its applications in image processing, natural language processing, computer graphics, and economics, as well as its relevance to brand-switching behavior. By placing our work within this, we highlight both the novelty and necessity of our method, emphasizing how it builds on and addresses limitations found in prior research.

Comment #3 *The manuscript does not include a dedicated discussion section, as it transitions*

directly from numerical experiment results to the conclusion. This omission limits the opportunity to critically interpret the findings, connect them to the broader literature, and explore their implications. For example, the results indicating shifts in market share due to liquor tax reforms and the launch of "Craft Boss" could be more deeply analysed to highlight their relevance to market strategy and policy-making. Furthermore, the discussion could address the robustness and potential limitations of the proposed method, such as the reliance on the cost matrix construction and assumptions about purchasing behaviour. Including a discussion section would allow the authors to better contextualise their contributions, acknowledge the study's limitations, and suggest practical applications and future research directions. This would significantly enhance the manuscript's clarity and impact.

Response: Thank you for your insightful comments. According to the reviewer's suggestion, we have added a discussion section in the revised manuscript. In this part, we discuss the implications of our results for market strategy and policy-making, highlighting the impact of liquor tax reforms and the launch of Craft Boss on market share shifts.

In addition, we address the potential limitations of our method, such as the reliance on the cost matrix and assumptions about purchasing behavior. While our model includes period-related and entropy regularization terms, the lack of customer IDs in the POS data prevented us from quantitatively assessing whether these regularization terms improve estimation accuracy. Future studies with customer-linked data are needed for rigorous quantitative validation.

Additionally, we acknowledge that our model does not account for individual preferences, such as brand loyalty. Future research should explore ways to incorporate customer-specific factors, even in settings with limited customer information.

Comment #4 *The conclusion section does not conform to academic standards as it predominantly reiterates details from the methodology and results without providing a concise and impactful summary. A well-structured academic conclusion should:*

1. *Summarise the Study in a Concise Manner – Provide a clear and succinct recap of the research objectives, methods, and key findings, avoiding technical details that belong in earlier sections.*

2. *Highlight the Significance of the Study – Emphasise the overarching contribution of the research to the field without introducing new information or results.*

3. *Provide Closure – Offer a final, thoughtful remark that underscores the importance of the findings in a broader context, leaving the reader with a sense of completion.*

Currently, the conclusion repeats details from the methodology and results rather than summarising the study holistically. To meet academic expectations, the authors should reframe this section to focus on the broader implications of their findings and provide a concise yet impactful closure.

Response: Thank you for your constructive feedback. According to the reviewer's suggestion, we have revised the conclusion to offer a succinct overview of our study's objectives, principal findings, and implications, rather than repeating detailed methods or results. We clarify how our approach can shed light on market shifts under privacy constraints and discuss future work on refining cost matrix construction and adding consumer-specific attributes like brand loyalty. We believe that this revision provides a clear sense of closure and underscores the broader significance of our findings.

References

[1] Amir Beck and Marc Teboulle. Mirror descent and nonlinear projected subgradient methods for convex optimization. *Operations Research Letters*, 31(3):167–175, 2003.

[2] Tomoaki Chiba, Hideitsu Hino, Shotaro Akaho, and Noboru Murata. Time-varying transition probability matrix estimation and its application to brand share analysis. *PLOS ONE*, 12(1):e0169981, 2017.
